# Semisynthesis and Antifeedant Activity of New Derivatives of a Dihydro-β-Agarofuran from *Parnassia wightiana*

**DOI:** 10.3390/ijms141019484

**Published:** 2013-09-26

**Authors:** Jiang-Jiang Tang, Fei-Yu Zhang, Dong-Mei Wang, Jun-Mian Tian, Shuai Dong, Jin-Ming Gao

**Affiliations:** 1 Shaanxi Engineering Center of Bioresource Chemistry & Sustainable Utilization, College of Science, Northwest A&F University, Yangling 712100, Shaanxi, China; E-Mails: tangjiang11@nwsuaf.edu.cn (J.-J.T.); feiyuzhang2013@outlook.com (F.-Y.Z.); tianjunmian@nwsuaf.edu.cn (J.-M.T.); phd@nwsuaf.edu.cn (S.D.); 2 College of Forestry, Northwest A&F University, Yangling 712100, Shaanxi, China; E-Mail: dmwli@nwsuaf.edu.cn

**Keywords:** sesquiterpene, agarofuran, derivatives, antifeedant activity

## Abstract

Five new derivatives (**2**–**6**) were semi-synthesized using compound **1**, a dihydro-β-agarofuran sesquiterpene with C-2 ketone obtained from *Parnassia wightiana*, as the starting material by acylation, oxidation, reduction, esterification, and amination, respectively. Structures of **2**–**6** were confirmed by 1D- and 2D-NMR and HR-ESI-MS spectra. In addition, antifeedant activities of these compounds (**1**–**6**) were tested against the 3rd-instar larvae of *Mythimna separata*. Antifeedant effects of compounds **2** and **4** were greater than the parent compound **1** whereas other compounds exhibited low to no feeding deterrent effects against third instar *M. separata* larvae in lab bioassays. Therefore, our results suggest that acylated and reduced derivatives at C-8 and C-2, respectively, of **1** may improve the antifeeding effect. This preliminary information will be useful in designing new insect control agents against *M. separata* and other important pests.

## 1. Introduction

The development of resistance to existing classes of pesticide and the increasing environmental pollution and toxicity generate a continuing need for the development of more active new classes of pest control agents against the target pests. During the last three decades, both synthetic and botanical pesticides from a variety of sources have been identified and developed, with most of the plant defensive chemicals discouraging insect herbivory, either by deterring feeding or by impairing larval growth, rather than by killing insects outright [1,2]. One of these pesticides structure is sesquiterpene polyesters with a dihydro-β-agarofuran skeleton such as Celangulin-V, which are the most widespread and characteristic group of secondary metabolites isolated mainly from Celastraceae family [3]. These compounds have attracted considerable attention from synthetic organic chemists and pharmacologists due to their complex structures and wide range of biological activities [4,5] including antifeedant activity, insecticidal activity, cytotoxicity [6,7], multidrug resistance (MDR) reversal activity [8–11], HIV inhibition [12] and antitumor activity [13]. Recently, Zhang and co-workers synthesized a series of ester [14], ether [15] and dimmer [16] derivatives using Celangulin-V as a lead compound, and found some potent insecticidal compounds.

**Figure 1:**
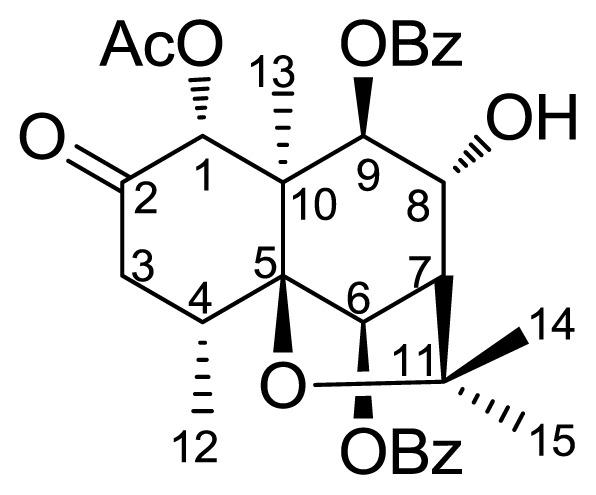
Structure of the natural sesquiterpene **1** from *P. wightiana.*

*Parnassia wightiana* Wall. (Family: Saxifragaceae) is a widely occurring perennial rhizomatous herb in East Asia, North American and Europe [17]. This plant was characterized by flavonoids, alkaloids and sesquiterpenoids. In our previous report [18], a new dihydro-β-agarofuran sesquiterpene with C-2 ketone (compound **1**, Figure 1) was isolated from this plant, showing significant cytotoxic activity against HepG2 (hepatocellular carcinoma) and MDA-10 (breast carcinoma) cells. To learn about the antifeedant activity of the natural sesquiterpene **1** and bioactivity changes resulting from altering the parent compound, **1** was used as the starting material and was transformed into derivatives **2**–**6** by acylation, oxidation, reduction, esterification and amination at C-2 or C-8 position of **1**. Herein, we describe semisynthesis and antifeedant activity of these new derivatives.

## 2. Results and Discussion

The synthetic pathways of derivatives **2**–**6** are shown in Scheme 1. All the structures of these derivatives were established on the basis of 1D- and 2D-NMR and HR-ESI-MS spectroscopic analysis (Figures S1–S19). NMR spectra are shown in Tables 1 and 2.

**Figure 2:**
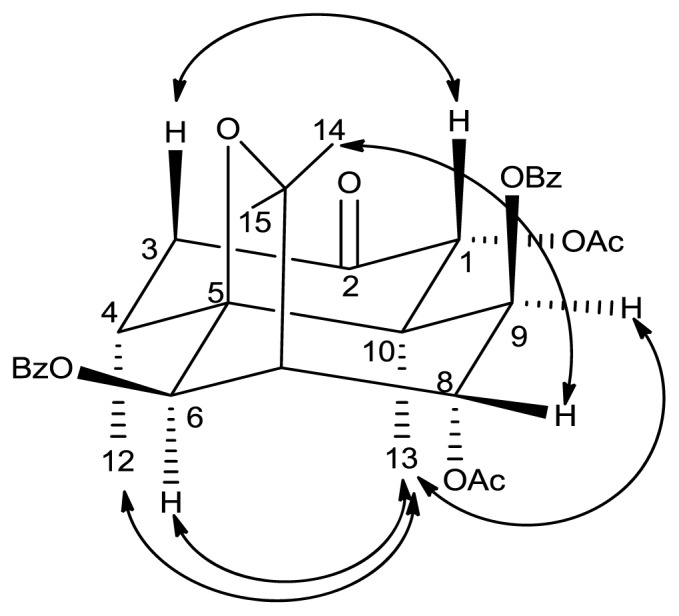
NOESY experiment of compound **2**.

Compound **2**, obtained through acylation [19] of **1** in the presence of triethylamine (Et_3_N) and catalytic dimethylaminopyridine (DMAP) in dry dichloromethane (DCM), showed an accurate [M + Na]^+^ ion at *m*/*z* 615.2219 in the HR-ESI-MS, corresponding to the molecular formula C_33_H_36_O_10_. In the ^1^H-NMR spectrum of **2**, the signal of H-8 for **1** at 4.45 ppm had moved to 5.39 ppm. Further assignments were made based on DEPT, HSQC, and HMBC experiments spectra. Structure, numbering and key NOESY correlations of **2** are shown in Figure 2.

Compound **3** was obtained through mild Dess-Martin oxidation at the 8-OH using 1,1,1-tris(acetoxy)-1,1-dihydro-1,2-benziodoxol-3-(1*H*)-one (DMP). Signal of the C-8 for **3** appears at δ 202.2, stating existence of a carbonyl group. Reduction with NaBH_4_ at the C-2 carbonyl of **1** at room temperature yielded mainly compound **4** with α-OH configuration at position C-2. This result was supported by the presence of coupling constant (*J* = 3.0 Hz) of H_ax_-1 through H_ex_-2 being located at chemical shift δ 5.56 ppm. This is due to that coupling constant of H_ax_ and H_ex_ is 2~3 Hz, which is smaller than that of H_ax_ and H_ax_ (*J* = 6~8 Hz).

Compounds **5** and **6** were obtained using esterification of **1** followed by amination. It is well-known that morpholine is one of the commonly used chemical groups because it plays an important role in medicinal chemistry and agrochemicals, such as morpholine derivatives Amorolfine [20] used as agricultural fungicides. To investigate whether hybrid of morpholine and compound **1** would display multiple activity or improved activity, we firstly prepared compound **5** through Yamaguchi esterification [21] at 8-OH of **1**, which involves the reaction of a 2-chloroacetic acid with 2,4,6-trichlorobenzoyl chloride to form the mixed anhydride, and upon reaction with an 8-OH in the presence of DMAP to produce the 2-chloroacetic ester derivative **5** in 85% yield. Then, amination of compound **5** with morpholine afforded the compound **6**.

Next, compound **1** and derivatives (**2**–**6**) was evaluated for their antifeedant activity against *M. separata* using a leaf disk choice bioassay [22]. The results are presented in Table 3. Some of the compounds caused more reduction in feeding of third instar larvae of *M. separata*, when given a choice between control and treated wheat leaf disks at 4 μg/cm^2^ at 24 and 48 h. Compounds **2** and **4** were respectively1.2 and 1.5 times more active as a feeding deterrent than the parent compound **1**. Other compounds caused 0–15% feeding deterrent effect. The results showed that compounds **2** and **4** were more active than the parent compound **1** at 24 h and 48 h but less potent than the positive control probenazole (100% feeding deterrent effect), an agrochemical used for the protection of rice plants [23]. The other compounds (**3**, **5** and **6**) showed weak or no feeding deterrent effect. Preliminary results suggested that acylated and reduced derivatives at C-8 and C-2, respectively, of **1** may improve the antifeedant effect, while oxidation, esterification and morpholine hybrids at C-8 of **1** may decrease the effect.

## 3. Experimental Section

### 3.1. General

NMR spectra were recorded on a Bruker Advance III 500 instrument (Bruker Daltonics Inc., Bremen, Germany) in CDCl_3_ with TMS as internal standard for protons and solvent signals as internal standard for carbon spectra. Chemical shift values are mentioned in δ (ppm) and coupling constants are given in Hz. Mass spectra (MS) were recorded on an ESI-esquire 3000 Bruker Daltonics instrument (Bruker Daltonics Inc., Bremen, Germany). HR-ESI-MS data were collected on Shimadzu liquid chromatography-mass spectrometry (LCMS)-iontrap (IT)-time of flight (TOF) (Shimadzu, Kyoto, Japan). Analytical thin-layer chromatography (TLC) was carried out on silica gel (Qingdao Marine Chemical, Ltd., Qingdao, China) plates, and spots were visualized by spraying with 5% H_2_SO_4_ in ethanol reagent (Sinopharm Chemical Reagent Co. Ltd., Shanghai, China) followed by heating at 120 ºC. Separation and purification were carried out by column chromatography on silica gel (200–300 mesh) (Qingdao Haiyang Chemical Group Co., Qingdao, China). Yields were not optimized. Solvents were dried by standard methods and distilled. Compound **1** (1α-acetoxy-6β,9β-dibenzyloxy- 8α-hydroxy-2-oxodihydro-β-agarofuran) was isolated from *P. wightiana*, and established on the basis of extensive spectroscopic analyses [18].

### 3.2. Semisynthesis of Derivatives (**2**–**6**)

Compound **2** (1α,8α-diacetoxy-6β,9β-dibenzyloxy-2-oxodihydro-β-agarofuran): To a stirred solution of compound **1** (22 mg, 0.04 mmol) in dichloromethane (DCM, 10 mL; Sinopharm Chemical Reagent Co.,Ltd, Shanghai, China) was added acetyl chloride (0.05 mL) and a catalytic amount of 4-(*N*,*N*′-dimethylamino)pyridine (DMAP) (Sinopharm Chemical Reagent Co. Ltd., Shanghai, China) at room temperature, and then the mixture was further stirred for 4 h with monitoring by TLC analysis. The reaction mixture was poured into ice water (10 mL) and extracted with ethyl acetate (3 × 10 mL). The combined organic layer was washed with NaHCO_3_ solution and dried over anhydrous Na_2_SO_4_. After evaporation in vacuum, the resulting solid was subjected to a silica-gel column chromatography with AcOEt/petroleum ether (1:6) as the eluent to give compound **2** in almost 65% yield. Yellow solid; AcO-1, BzO-6, BzO-9 was certified by ^1^H NMR δ: 1.71 (s, 3H, AcO-1), 8.02 (d, 2H, *J* = 7.7 Hz, BzO-6), 7.50 (t, 2H, *J* = 7.7 Hz, BzO-6), 7.62 (t, 1H, *J* = 7.7 Hz, BzO-6), 8.04 (t, 2H, *J* = 7.7 Hz, BzO-9), 7.46 (t, 2H, *J* = 7.7 Hz, BzO-9), 7.58 (t, 1H, *J* = 7.7 Hz, BzO-6);^13^C NMR δ: 169.8 (1C), 164.6 (1C), 20.0 (1C), 133.6 (1C), 129.9 (2C), 128.6 (2C), 128.8 (1C), 165.3 (1C), 133.7 (1C), 129.5 (3C), 128.5 (2C), other data see Tables 1 and 2; ESI-MS *m*/*z*: 593.96 [M + H]^+^, 615.94 [M + Na]^+^; HR-ESI-MS *m/z*: 615.2219 [M + Na]^+^ (calcd. for C_33_H_36_O_10_Na, 615.2201).

Compound **3** (1α-acetoxy-6β,9β-dibenzyloxy-2,8-dioxodihydro-β-agarofuran): To a stirred solution of compound **1** (11 mg, 0.02 mmol) in DCM (3 mL) was added DMP (0.024 mmol; J & K Scientific Ltd., Beijing, China) at room temperature, and then the mixture was further stirred for 3 h, with monitoring by TLC analysis. The reaction mixture was poured into ice water (10 mL) and extracted with AcOEt (3 × 10 mL; Sinopharm Chemical Reagent Co. Ltd., Shanghai, China). The combined organic layer was washed with NaHCO_3_ solution and dried over anhydrous Na_2_SO_4_. After evaporation in vacuum, the resulting solid was subjected to a silica-gel column chromatography with AcOEt/petroleum ether (1/6) as the eluent to give compound **3** in 90% yields. White solid; AcO-1, BzO-6, BzO-9 was certified by ^1^H NMR δ: 1.67 (s, 3H, AcO-1), 8.06 (d, 2H, *J* = 7.7 Hz, BzO-6), 7.52 (t, 2H, J = 7.7 Hz, BzO-6), 7.65 (t, 1H, *J* = 7.7 Hz, BzO-6), 8.00 (t, 2H, *J* = 7.7 Hz, BzO-9), 7.43 (t, 2H, *J* = 7.7 Hz, BzO-9), 7.58 (t, 1H, *J* = 7.7 Hz, BzO-6);^13^C NMR δ: 169.3 (1C), 20.2 (1C), 164.5 (1C), 133.9 (1C), 130.0 (2C), 128.6 (2C), 128.9 (1C), 128.3 (1C), 164.8 (1C), 134.1 (1C), 129.7 (1C), 128.8 (2C), 128.9 (1C), other data see Tables 1 and 2; ESI-MS *m*/*z*: 565.83 [M + NH_4_]^+^, 571.22 [M + Na]^+^; HR-ESI-MS *m/z*: 571.1962 [M + Na]^+^ (calcd. for C_31_H_32_O_9_Na, 571.1939).

Compound **4** (1α-acetoxy-6β,9β-dibenzyloxy-2α,8α-dihydroxydihydro-β-agarofuran): To a stirred solution of compound **1** (22 mg, 0.04 mmol) in anhydrous methanol (10 mL) was added NaBH_4_ (10 mg, 0.2 mmol) in 10 min at room temperature, and then the mixture was further stirred for 1 h with monitoring by TLC analysis. The reaction mixture was extracted with AcOEt (3 × 10 mL). The combined organic layer was washed with HCl (1 mol/L) solution to pH = 7 and dried over anhydrous Na_2_SO_4_. After evaporation in vacuum, the resulting solid was subjected to a silica-gel column chromatography with AcOEt/petroleum ether (1:3) as the eluent to give compound **4** in almost 85% yield. White solid; AcO-1, BzO-6, BzO-9 was certified by ^1^H NMR δ: 1.73 (s, 3H, AcO-1), 8.01 (d, 2H, *J* = 7.7 Hz, BzO-6), 7.53 (t, 2H, *J* = 7.7 Hz, BzO-6), 7.65 (t, 1H, *J* = 7.7 Hz, BzO-6), 8.10 (t, 2H, *J* = 7.7 Hz, BzO-9), 7.48 (t, 2H, *J* = 7.7 Hz, BzO-9), 7.60 (t, 1H, *J* = 7.7 Hz, BzO-6); ^13^C NMR δ: 170.0 (1C), 20.8 (1C), 165.5 (1C), 133.3 (1C), 129.7 (1C), 128.8 (1C), 130.2 (1C), 128.7 (1C), 129.6 (1C), 165.6 (1C), 133.3 (1C), 130.0 (1C), 128.6 (1C), 128.4 (1C), 128.6 (1C), 130.1 (1C), other data see Tables 1 and 2; ESI-MS *m*/*z*: 552.77 [M + H]^+^, 575.12 [M + Na]^+^; HR-ESI-MS *m/z*: 575.2273 [M + Na]^+^ (calcd. for C_31_H_36_O_9_Na, 575.2252).

Compound **5** (1α-acetoxy-6β,9β-dibenzyloxy-8α-chloracetyl-2-oxodihydro-β-agarofuran): To a solution of chloroactic acid (4 mg, 0.04 mmol) in toluene (0.2 mL) at room temperature were added Et_3_N (11 μL, 0.08 mmol) and 2,4,6-trichlorobrnzoyl chloride (6.4 μL, 0.05 mmol). The resulting mixture was stirred for 1 h before a solution of compound **1** (66 mg, 0.12 mmol) and DMAP (6.3 mg, 0.05 mmol) in toluene (0.15 mL) added. The resulting mixture was heated to 80 ºC and stirred for 24 h, then it was quenched with a NaHCO_3_ (1 mL). The layers were separated and the aqueous layer was extracted with EtOAc (3 × 10 mL). The combined organic layers were washed with brine (3 mL), dried (Na_2_SO_4_) and concertrated in vacuum. The resulting solid was subjected to a silica-gel column chromatography with AcOEt/petroleum ether (1:10) as the eluent to give compound **5** in almost 85% yield. White solid; AcO-1, BzO-6, BzO-9 was certified by ^1^H NMR δ: 1.72 (s, 3H, AcO-1), 8.02 (d, 2H, *J* = 7.7 Hz, BzO-6), 7.50 (t, 2H, *J* = 7.7 Hz, BzO-6), 7.63 (t, 1H, *J* = 7.7 Hz, BzO-6), 8.04 (t, 2H, *J* = 7.7 Hz, BzO-9), 7.47 (t, 2H, *J* = 7.7 Hz, BzO-9), 7.60 (t, 1H, *J* = 7.7 Hz, BzO-6); ^13^C NMR δ: 169.3 (1C), 20.0 (1C), 164.6 (1C), 133.8 (1C), 129.6 (2C), 128.7 (2C), 129.9 (1C), 166.0 (1C), 133.1 (1C), 128.8 (2C), 128.7 (2C), 128.2 (1C), other data see Tables 1 and 2; ESI-MS *m*/*z*: 626.71 [M + H]^+^, 649.01 [M + Na]^+^; HR-ESI-MS *m/z*: 649.1829 [M + Na]^+^ (calcd. for C_33_H_35_O_10_ClNa, 649.1811).

Compound **6** (1α-acetoxy-6β,9β-dibenzyloxy-8α-morpholinoacetoxy-2-oxodihydro-β-agarofuran): To a stirred solution of compound **5** (25 mg, 0.04 mmol) in DCM (10 mL) was added morpholine (0.05 mL) at room temperature, and then the mixture was further stirred for 20 h, with monitoring by TLC analysis. The reaction mixture was poured into ice water (10 mL) and extracted with ethyl acetate (3 × 10 mL). The combined organic layer was washed with NaHCO_3_ solution and dried over anhydrous Na_2_SO_4_. After evaporation in vacuum, the resulting solid was subjected to a silica-gel column chromatography with methanol/chloroform (1:50) as the eluent to give compound **6** in almost 50% yield. Yellow solid; AcO-1, BzO-6, BzO-9 was certified by ^1^H NMR δ: 1.71 (s, 3H, AcO-1), 8.02 (d, 2H, *J* = 7.7 Hz, BzO-6), 7.49 (t, 2H, *J* = 7.7 Hz, BzO-6), 7.62 (t, 1H, *J* = 7.7 Hz, BzO-6), 8.02 (t, 2H, *J* = 7.7 Hz, BzO-9), 7.47 (t, 2H, *J* = 7.7 Hz, BzO-9), 7.60 (t, 1H, *J* = 7.7 Hz, BzO-6); ^13^C NMR δ: 169.3 (1C), 20.0 (1C), 164.6 (1C), 133.9 (1C), 129.5 (2C), 128.8 (2C), 129.9 (1C), 168.9 (1C), 133.8 (1C), 129.4 (2C), 128.7 (2C), 128.4 (1C), other data see Tables 1 and 2; HR-ESI-MS *m/z*: 678.2915 [M + H]^+^ (calcd. for C_37_H_44_NO_11_, 678.2909).

### 3.3. Antifeedant Test

Third instar larvae of *M. separata* Walker (armyworm), provided by the Institute of Pesticides, Northwest A & F University, were used as test insect for evaluation of antifeedant activity using a leaf disk choice bioassay [22]. *M. separata* was continuously maintained on a Petri dish (Ø 10 cm) in a growth chamber in our laboratory at a temperature of 27 ºC and 60% relative humidity under a 16L:8D photoperiod. Leaf discs (0.5 × 0.5 cm) were cut from wheat leaves (*Triticum aestivum*; Poaceae). Control wheat leaf discs were painted on each side with 10 μL of the carrier solvent (acetone; Sinopharm Chemical Reagent Co. Ltd., Shanghai, China), and test wheat leaf discs with the same amount of the test solution at 100 μg/mL (4 μg/cm^2^ of the final concentration). After the solvent had evaporated, it was treated and control discs were placed in each compartment of a plastic assay tray. Third instar larvae (weight 13–16 mg) starved for 5–7 h, was introduced gently into the center of each compartment. The number of larvae was 20 per treatment, and three replications were used in each experiment. Probenazole (J & K Scientific Ltd., Beijing, China) was used as a positive control. The amount of leaf area consumed at 24 and 48 h was estimated using squared graph paper. The antifeeding rate (%) was calculated using the formula [(C − T)/(C + T)] × 100, where C and T are areas consumed of the control and treated leaf disks, respectively. Experiment was repeated twice.

## 4. Conclusions

In conclusion, five new dihydro-β-agarofuran sesquiterpenes were semisynthesized using a dihydro-β-agarofuran with C-2 carbonyl (compound **1**) from *P. wightiana* as a starting material. Some sesquiterpenes showed certain antifeedant activity, and structure-activity relationships suggested that the antifeedant effects of the derivatives depend on the position of hydroxyl and acetoxyl substituents at C-8 and C-2 positions (compounds **2** and **4**). This will be helpful in designing new dihydro-β-agarofuran insect control agents against *M. separata* and other important pests.

## Supplementary Information



## Figures and Tables

**Scheme 1:**
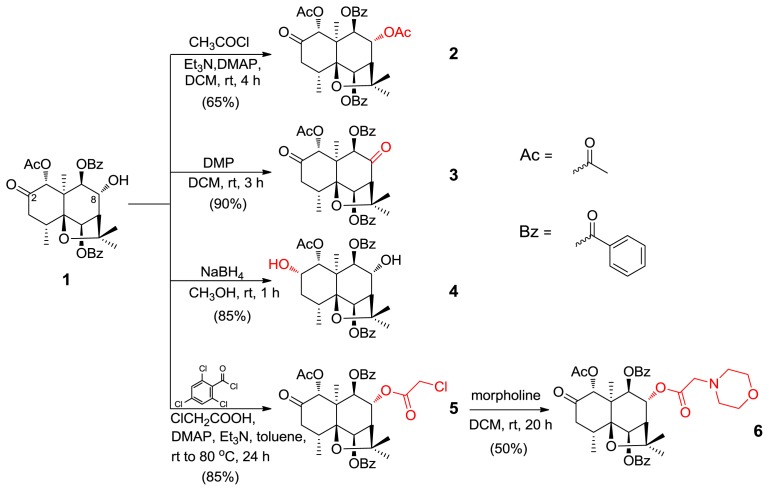
Semisynthesis of derivatives **2**–**6** from **1**. 4-dimethylaminopyridine (DMAP), 1,1,1-tris(acetoxy)-1,1-dihydro-1,2-benziodoxol-3-(1*H*)-one (DMP), dichloromethane (DCM).

**Table 1:** ^1^H NMR spectroscopic data of compound **2**–**6** (500 MHz, CDCl_3_) a.

Position	2	3	4	5	6
H-1	5.95 (s)	5.96 (s)	5.56 (d, 3.0)	5.95 (s)	5.95 (s)
H-2			2.09 (d, 3.0)		
H-3α	3.39 (dd, 12.5, 7.5)	3.40 (dd, 13, 7.5)	3.73 (m)	3.40 (dd, 12.8, 7.5)	3.40 (dd, 12.5, 7.5)
H-3β	2.30 (dd, 12.5, 1.5)	2.37 (d, 13)	2.55 (m)	2.32 (d, 13)	2.32 (d, 13)
H-4	3.02 (m)	3.09 (m)	2.67 (m)	3.05 (m)	3.03 (m)
H-6	6.04 (s)	5.89 (1H,s)	6.30 (s)	5.99 (s)	5.99 (s)
H-7	2.76 (d, 3.3)	3.24 (1H,s)	2.65 (d, 3.5)	2.84 (d, 4.0)	2.72 (d, 3.5)
H-8	5.39 (d, 3.3)		4.99 (d, 3.3)	5.17 (d, 3.3)	5.08 (d, 3.3)
H-9	5.09 (s)	5.49 (s)	4.47 (s)	5.52 (s)	5.43 (s)
H-12	1.05 (d, 7.5)	1.05 (d, 7.5)	1.36 (d, 8.0)	1.06 (d, 7.0)	1.05 (d, 6.5)
H-13	1.45 (s)	1.46 (3H,s)	1.45 (s)	1.45 (s)	1.49 (s)
H-14	1.57 (s)	1.54 (3H,s)	1.56 (s)	1.57 (s)	1.56 (s)
H-15	2.29 (s)	1.62 (3H,s)	1.62 (s)	1.61 (s)	1.62 (s)
AcO-8 (C*H*_3_)	1.61 (s)				
RC*H*_2_OCO-8^b^				4.35 (s)	3.46 (s)
N(CH_2_C*H*_2_)_2_O					2.78 (m)
N(CH_2_C*H*_2_)_2_O					3.57 (m)

a Data for additional ester groups are provided in the Experimental Section.

b For compound **5**, *R* = Cl; for compound **6**, *R* = N(CH_2_CH_2_)_2_O. Data are based on DEPT, HSQC, and HMBC experiments.

**Table 2:** ^13^C NMR Spectroscopic data of compound **2–6** (500 MHz, CDCl_3_) a.

Position	2	3	4	5	6
C-1	77.4	76.7	74.9	77.5	77.4
C-2	204.3	203.5	70.8	204.2	204.3
C-3	43.9	44.0	34.1	43.9	43.9
C-4	38.7	38.2	34.0	38.7	38.7
C-5	89.5	91.2	90.8	89.4	89.5
C-6	76.1	70.8	76.2	76.3	76.0
C-7	53.1	74.0	55.7	52.9	53.2
C-8	75.9	202.2	74.6	75.9	77.2
C-9	77.2	65.35	80.4	76.5	76.3
C-10	55.0	51.9	55.6	55.1	59.4
C-11	82.9	84.1	81.7	82.9	82.9
C-12	18.1	17.8	18.7	18.1	14.2
C-13	20.2	19.9	19.4	20.3	20.3
C-14	31.0	21.9	25.7	25.5	25.5
C-15	25.6	27.3	29.7	31.0	31.0
AcO-8	21.2, 169.3				
R*C*H_2_O*C*O-8 b				41.0, 172.6	55.0, 165.3
N(*C*H_2_CH_2_)_2_O					53.3
N(CH_2_*C*H_2_)_2_O					67.0

a Data for additional ester groups are provided in the Experimental Section.

b For compound **5**, *R* = Cl; for compound **6**, *R* = N(CH_2_CH_2_)_2_O. Data are based on DEPT, HSQC, and HMBC experiments.

**Table 3:** Antifeedant effects of compounds (**1**–**6**) against 3^rd^-instar larvae of *M. separata*^a^.

Entry	24 h-Antifeedant rate (%)	48 h-Antifeedant rate (%)
control	0	0
**1**	55	45
**2**	65	50
**3**	15	12.5
**4**	80	72.5
**5**	0	0
**6**	0	0
Probenazole	100	97.50

a Antifeedant effects of compound **1**, derivatives and probenazole (positive control) were measured at 4 μg/cm^2^ in acetone. Three replications (*N* = 20 larvae/treatment, the weight of larva was 13–16 mg) were used in each experiment and experiment was repeated twice. Means were reproducible with deviation less than ± 15%.
