# Supplementary material for: Semisynthesis and Antifeedant Activity of New Derivatives of a Dihydro-β-Agarofuran from Parnassia wightiana
Source: Int J Mol Sci. 2013 Sep 26;14(10):19484–93. doi: 10.3390/ijms141019484 (PMC3821568; doi:10.3390/ijms141019484)
Supplement: Supplementary file 1 [file ijms-14-19484-s001.pdf]

Supplementary Information

Figure 1. ^{1}H NMR (500 MHz, $CDCl_3$) spectra of compound **2**.

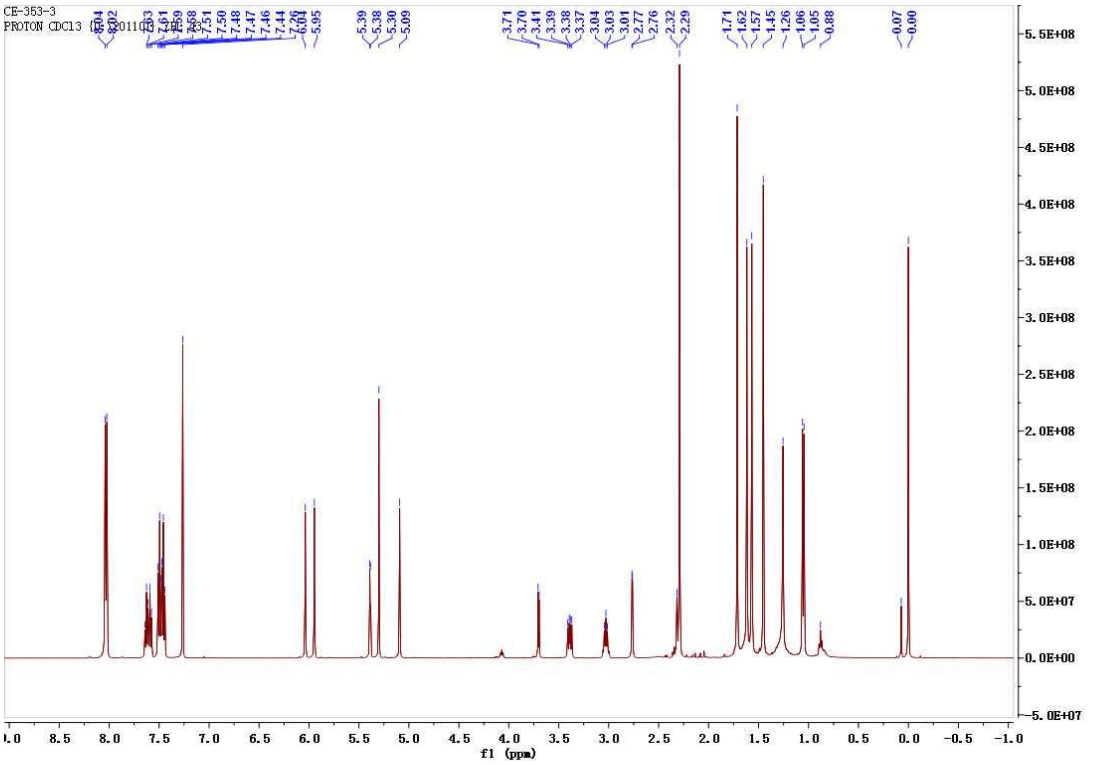

Figure 2. ^{13}C NMR (500 MHz, $CDCl_3$) spectra of compound **2**.

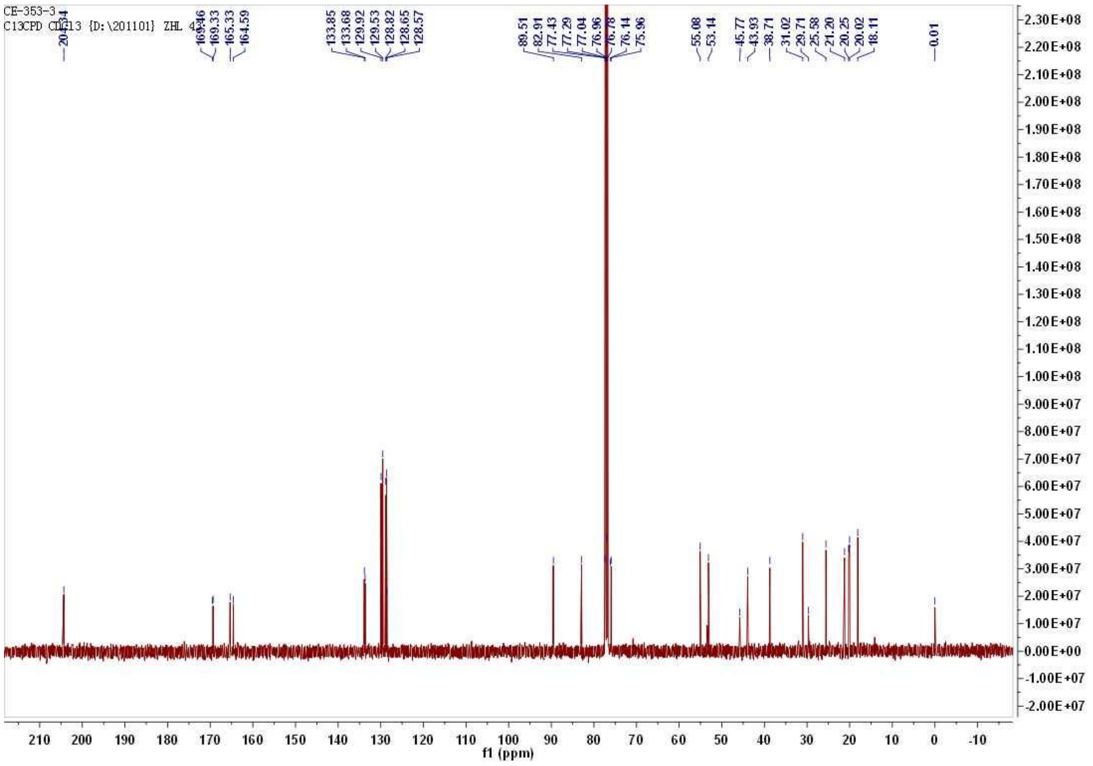

Figure 3. ^{1}H-^{1}H COSY (400 MHz, $CDCl_3$) spectra of compound **2**.

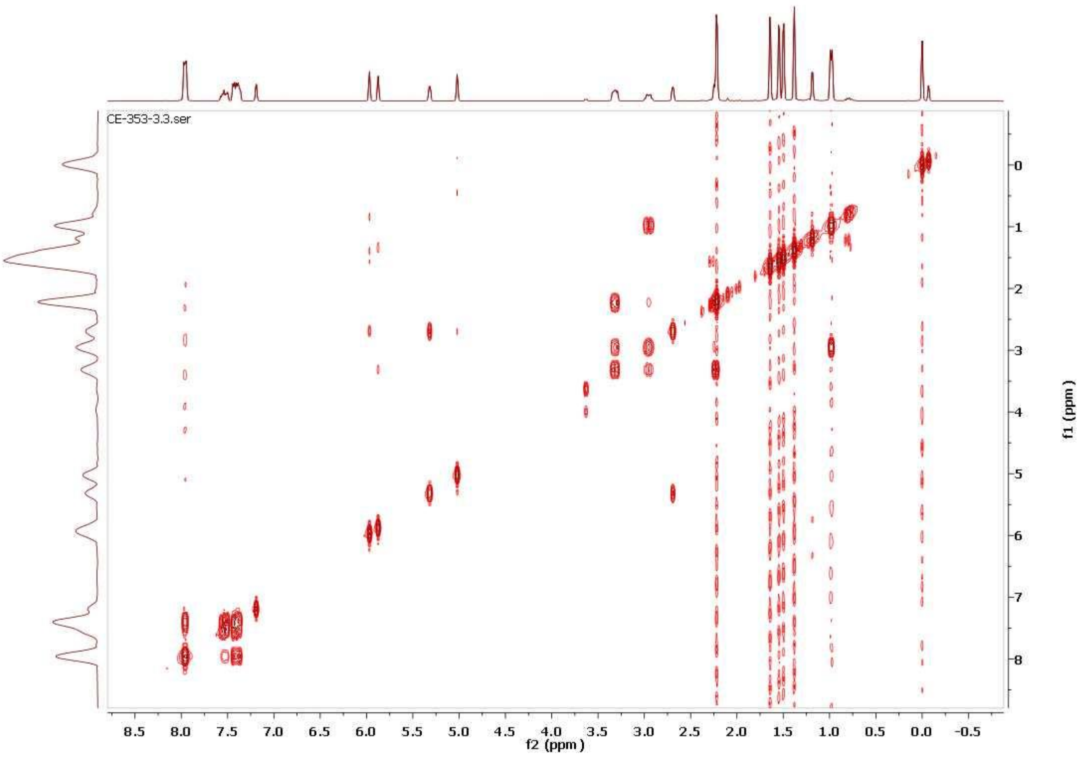

Figure 4. HMBC (400 MHz, $CDCl_3$) spectra of compound **2**.

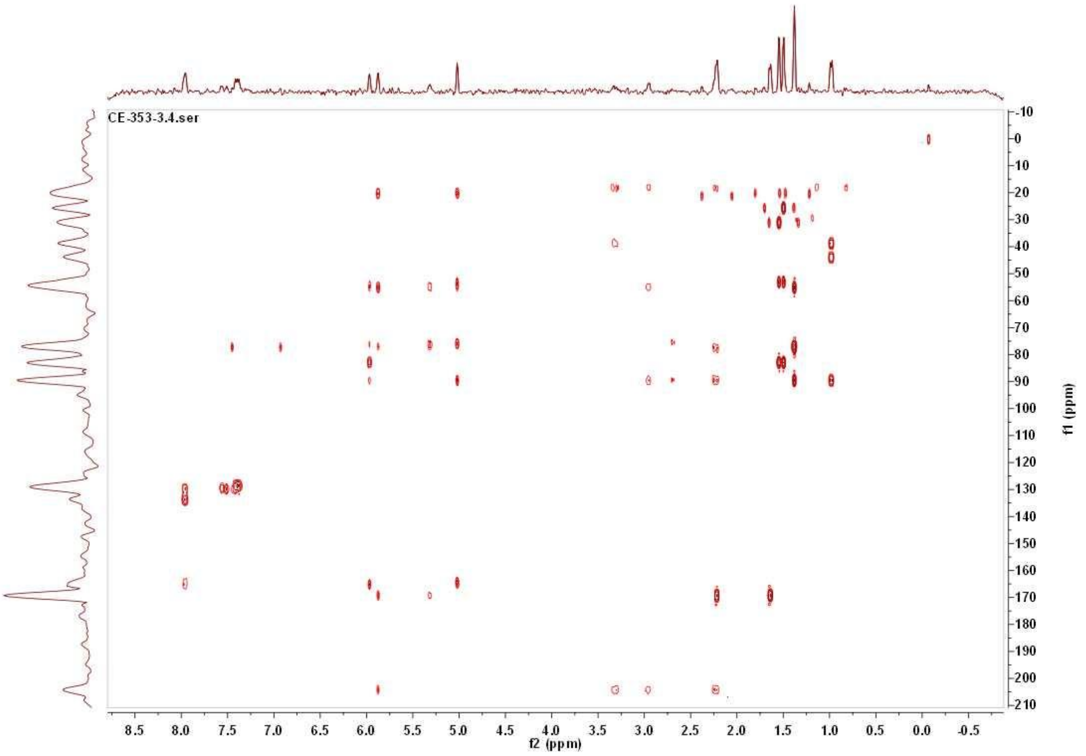

Figure 5. HSQC (400 MHz, $CDCl_3$) spectra of compound **2**.

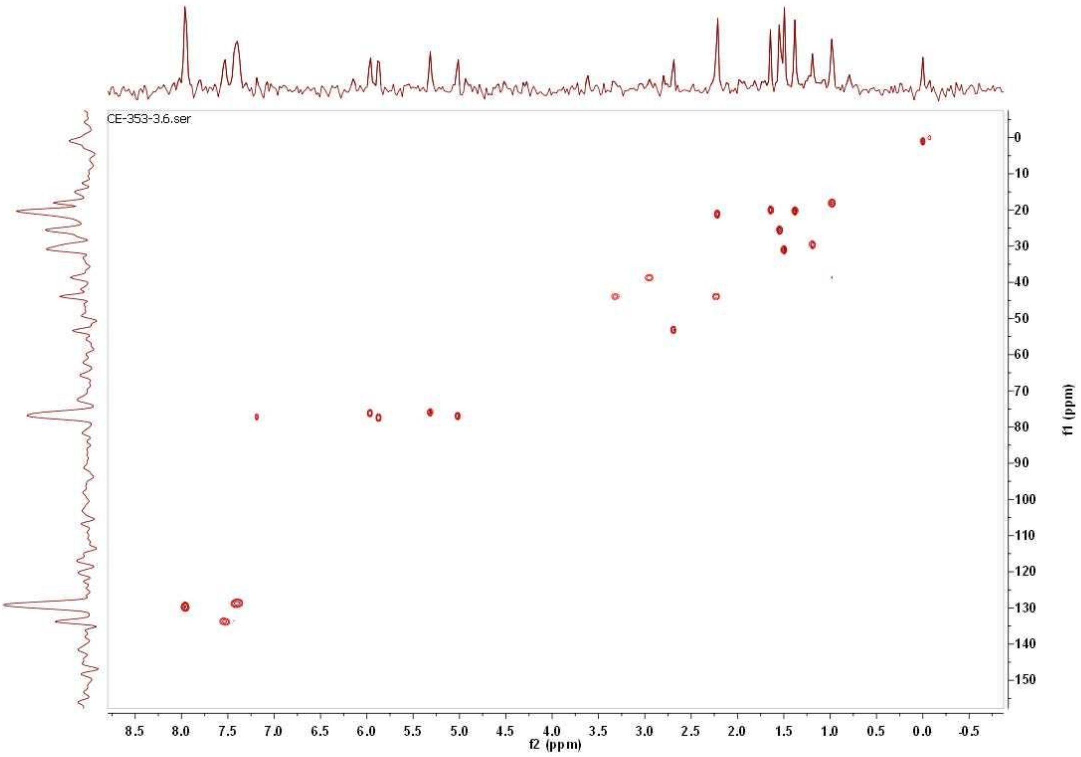

Figure 6. NOESY (400 MHz, $CDCl_3$) spectra of compound **2**.

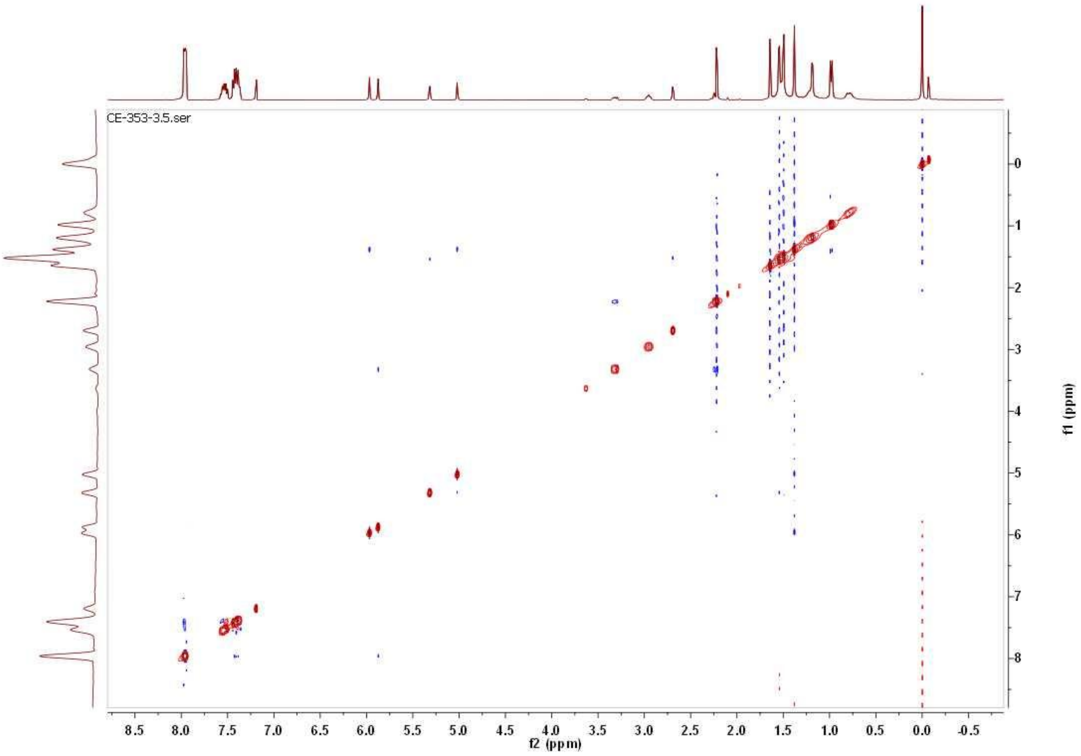

Figure 7. HRESIMS spectra of Compound **2**.

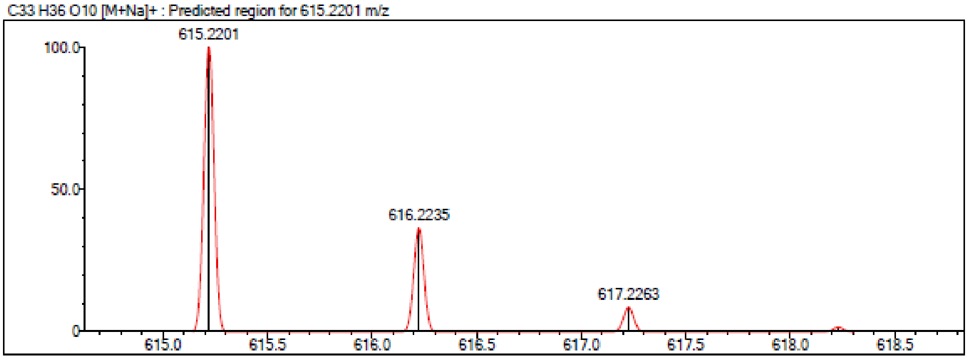

Rank	Score	Ion	Formula (M)	Pred. m/z	Meas. m/z	Df. (mDa)	Df. (ppm)	Iso	DBE
1	0.00	[M+Na]+	C33 H36 O10	615.2201	615.2219	1.8	2.93	0.00	16.0

Figure 8. ^{1}H NMR (500 MHz, $CDCl_3$) spectra of compound **3**.

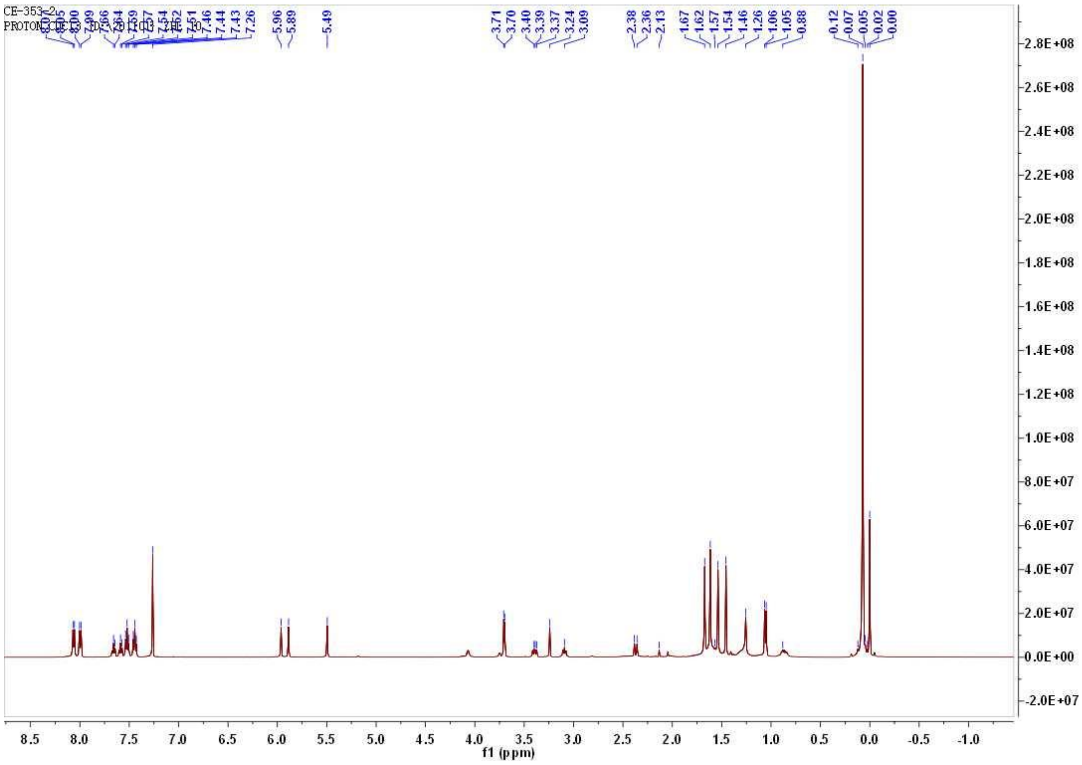

Figure 9. ^{13}C NMR (500 MHz, $CDCl_3$) spectra of compoun **3**.

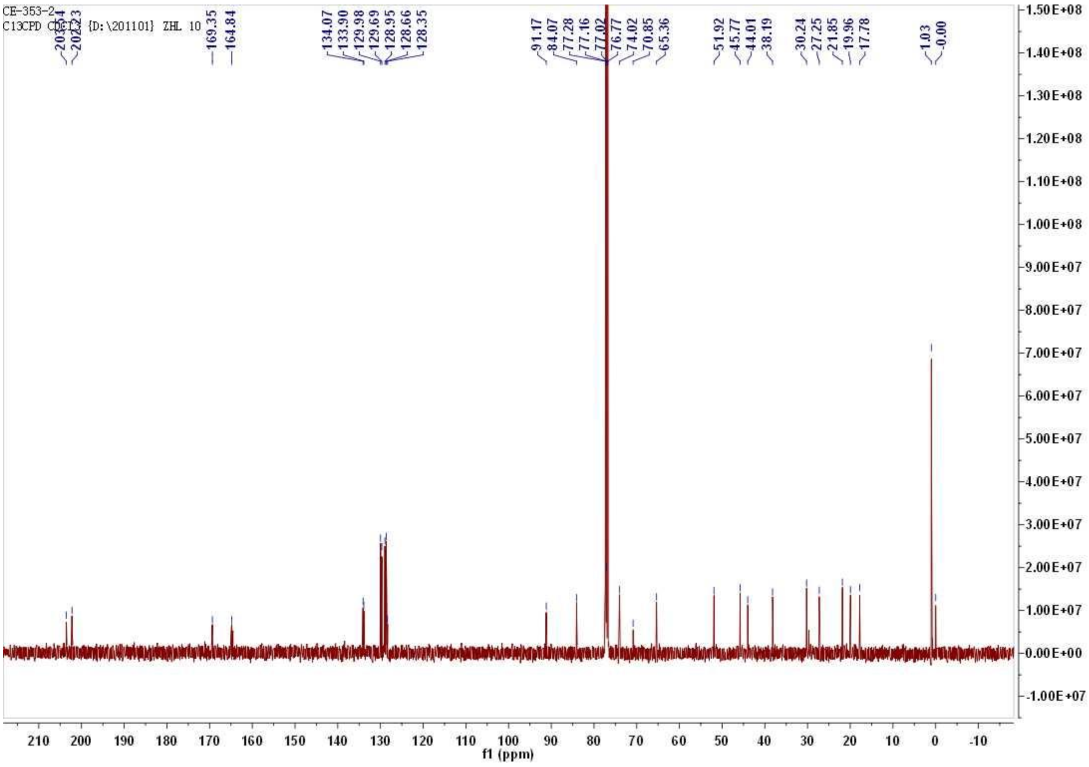

Figure 10. HRESIMS spectra of Compound **3**.

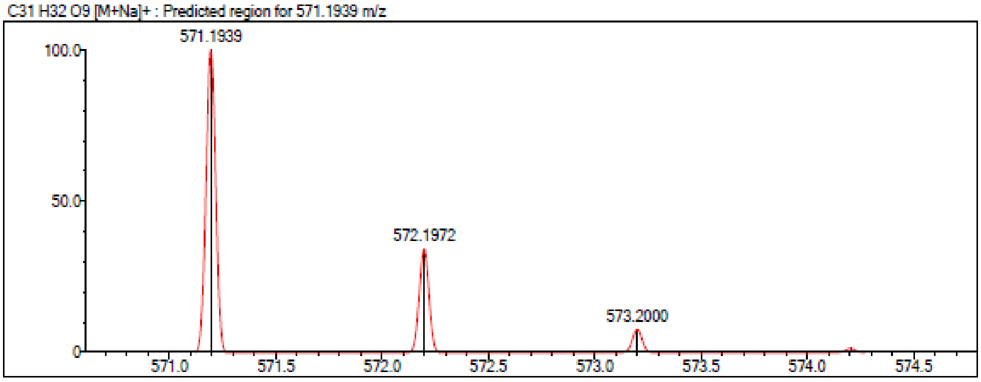

Rank	Score	Ion	Formula (M)	Pred. m/z	Meas. m/z	Df. (mDa)	Df. (ppm)	Iso	DBE
1	73.39	[M+Na]+	C31 H32 O9	571.1939	571.1962	2.3	4.03	79.40	16.0

Figure 11. ^{1}H NMR (500 MHz, $CDCl_3$) spectra of compound **4**.

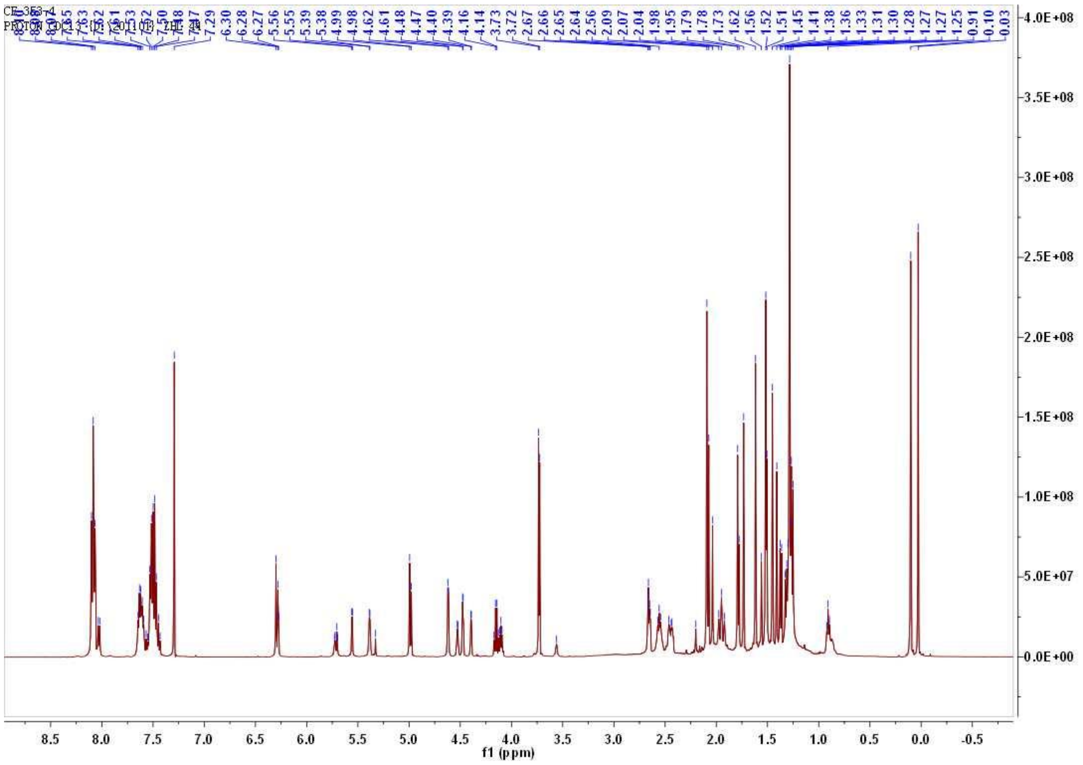

Figure 12. ^{13}C NMR (500 MHz, $CDCl_3$) spectra of compoun **4**.

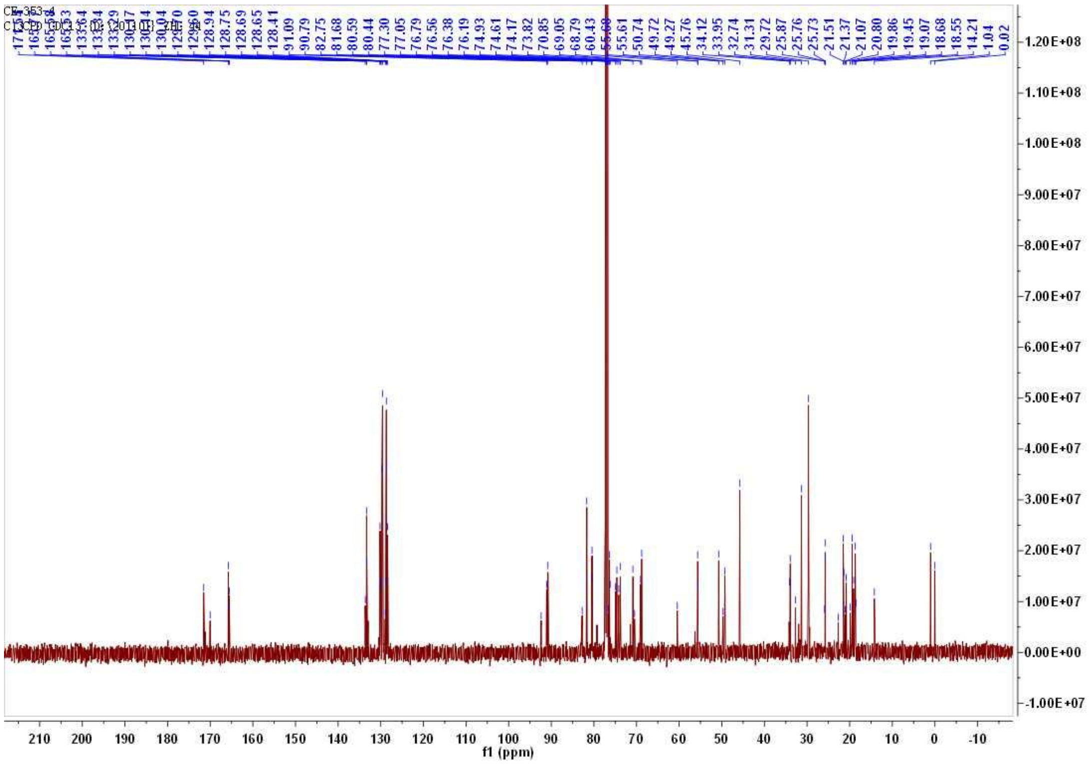

Figure 13. HRESIMS spectra of Compound **4**.

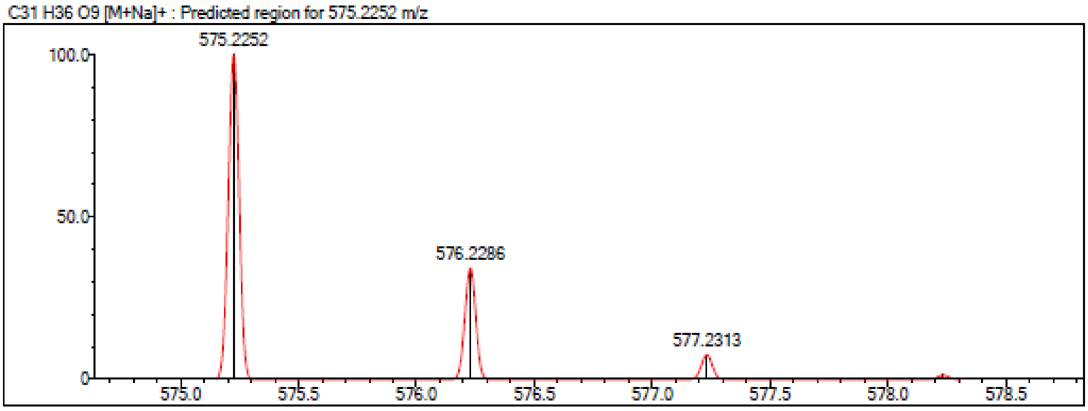

Rank	Score	Ion	Formula (M)	Pred. m/z	Meas. m/z	Df. (mDa)	Df. (ppm)	Iso	DBE
1	88.48	[M+Na]+	C31 H36 O9	575.2252	575.2273	2.1	3.65	94.75	14.0

Figure 14. ^{1}H NMR (500 MHz, $CDCl_3$) spectra of compound **5**.

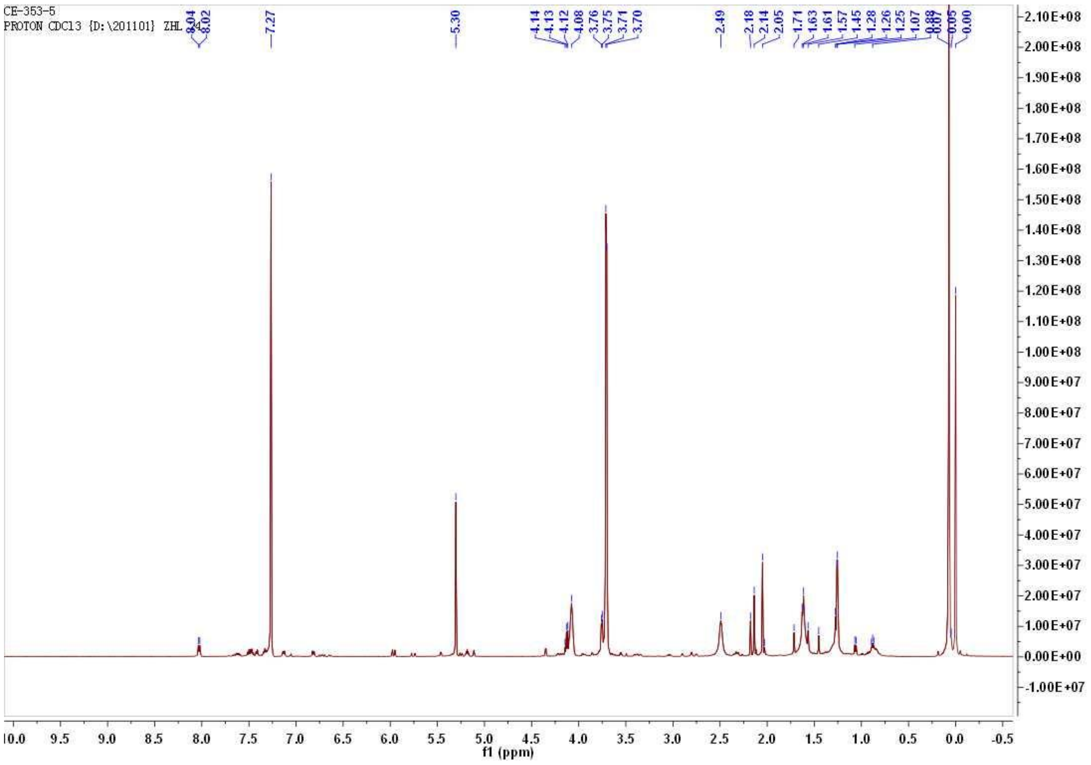

Figure 15. ^{13}C NMR (500 MHz, $CDCl_3$) spectra of compound **5**.

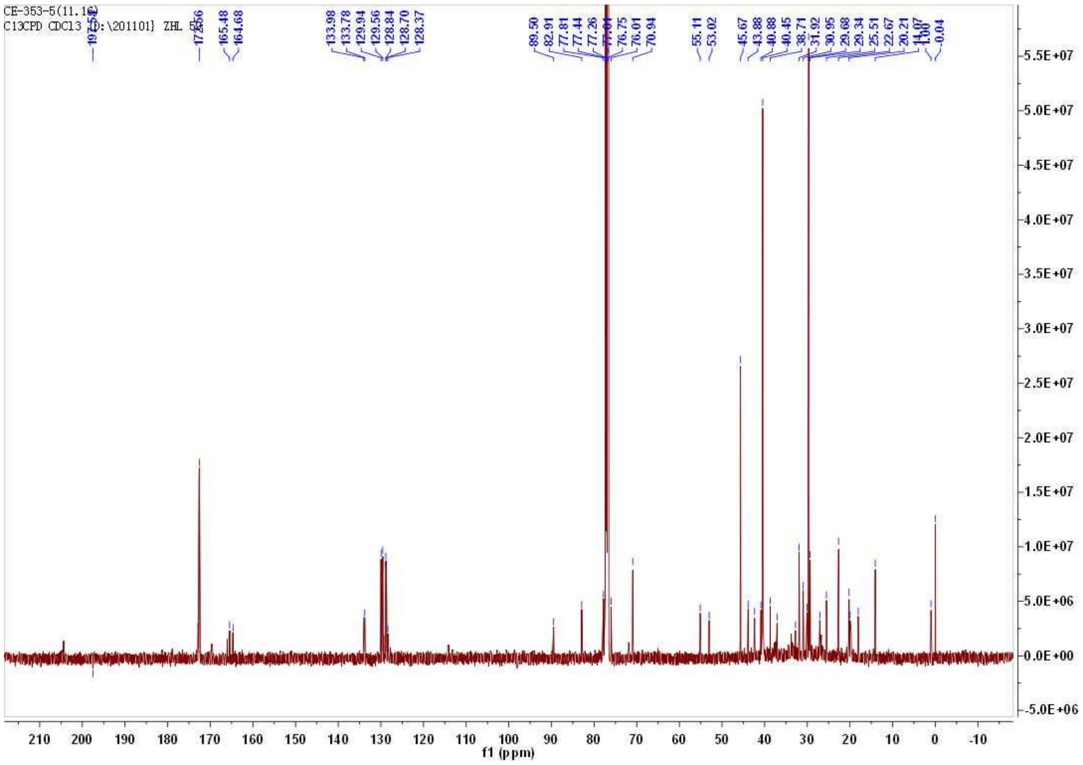

Figure 16. HRESIMS spectra of Compound **5**.

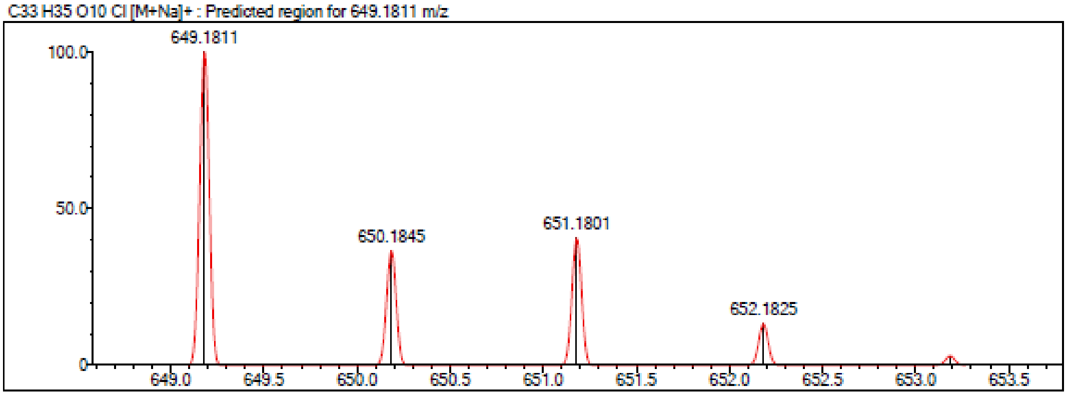

Rank	Score	Ion	Formula (M)	Pred. m/z	Meas. m/z	Df. (mDa)	Df. (ppm)	Iso	DBE
8	0.00	[M+Na]+	C33 H35 O10 Cl	649.1811	649.1829	1.8	2.77	0.00	16.0

Figure 17. ^{1}H NMR (500 MHz, $CDCl_3$) spectra of compound **6**.

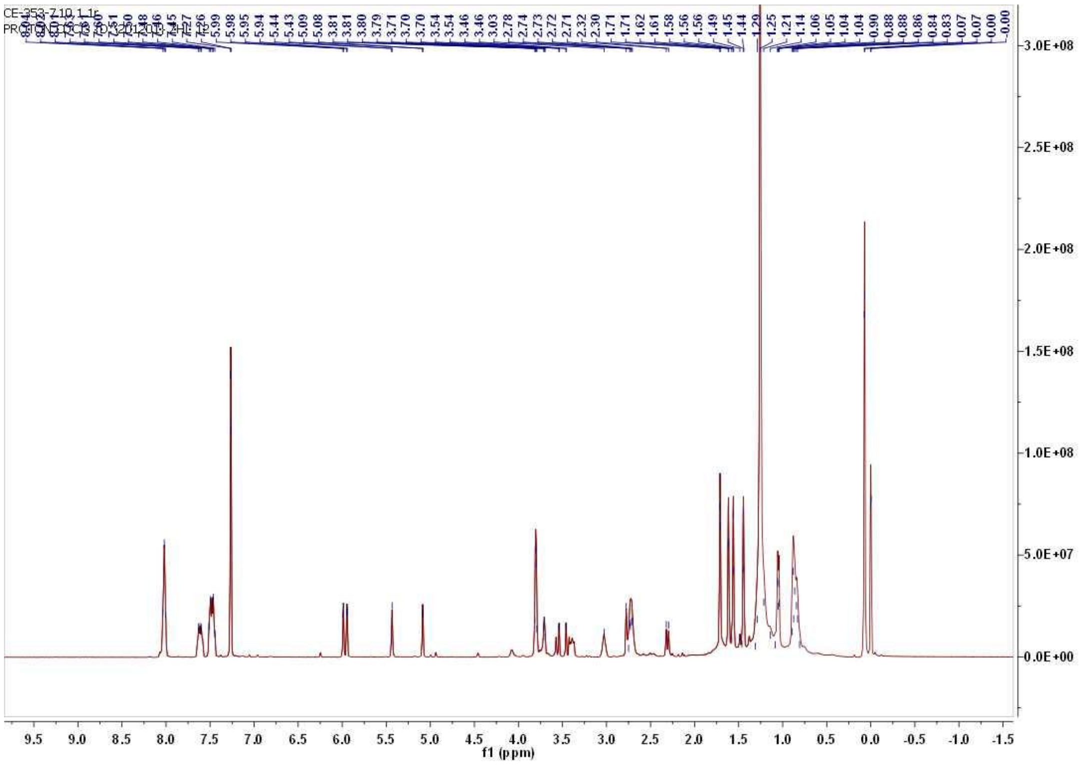

Figure 18. ^{13}C NMR (500 MHz, $CDCl_3$) spectra of compound **6**.

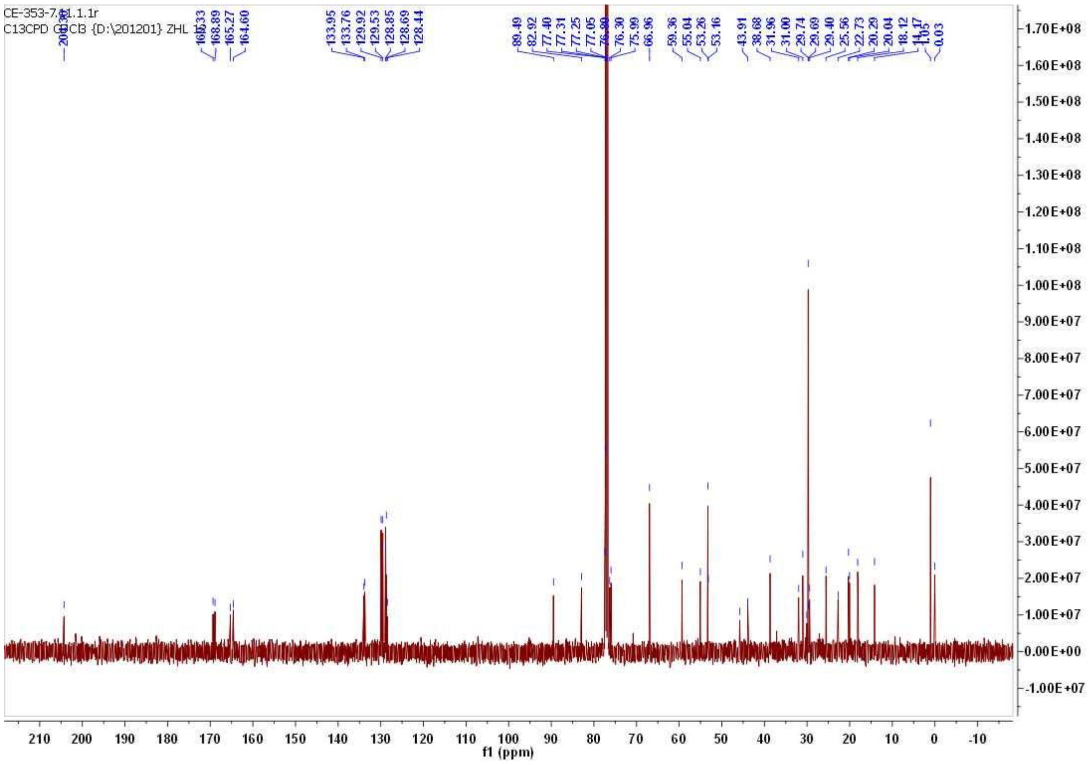

Figure 19. HRESIMS spectra of Compound **6**.

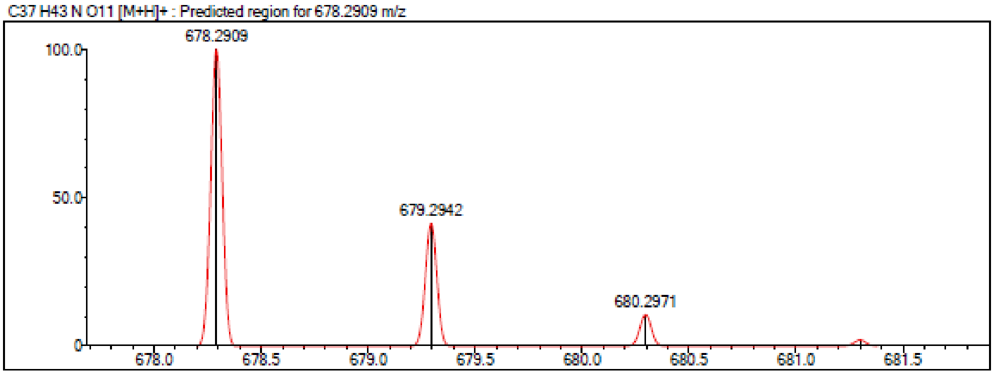

Rank	Score	Ion	Formula (M)	Pred. m/z	Meas. m/z	Df. (mDa)	Df. (ppm)	Iso	DBE
1	96.77	[M+H]+	C37 H43 N O11	678.2909	678.2915	0.6	0.88	96.77	17.0

© 2013 by the authors; licensee MDPI, Basel, Switzerland. This article is an open access article distributed under the terms and conditions of the Creative Commons Attribution license (http://creativecommons.org/licenses/by/3.0/).
